# Retraction: Queen cells acceptance rate and royal jelly production in worker honey bees of two *Apis mellifera* races

**DOI:** 10.1371/journal.pone.0277690

**Published:** 2022-11-16

**Authors:** The PLOS ONE Editors

The *PLOS ONE* Editors retract this article [1, 2] because it was identified as one of a series of submissions for which we have concerns about authorship, competing interests, and peer review. We regret that the issues were not addressed prior to the article’s publication.

MEAM agreed with retraction. KAK, HAG, ZA, and MAAEN either did not respond directly or could not be reached.
